# The experiences of women from culturally and linguistically diverse backgrounds with gestational diabetes mellitus: A mixed methods systematic review

**DOI:** 10.1002/edm2.421

**Published:** 2023-05-04

**Authors:** Claire Haigh, Hiu Wing Rachel Lau, Tessa Weir, Sarah Glastras

**Affiliations:** ^1^ Kolling Institute and North Precinct The University of Sydney Sydney New South Wales Australia; ^2^ Department of Diabetes, Endocrinology & Metabolism Royal North Shore Hospital St Leonards New South Wales Australia

**Keywords:** culturally and linguistically diverse, experience, gestational diabetes mellitus, mixed‐methods, systematic review

## Abstract

**Background:**

Gestational diabetes mellitus (GDM) is experienced at a higher rate in women from culturally and linguistically diverse (CALD) backgrounds. The aim of this systematic review is to describe the experiences of women with GDM from CALD backgrounds and compare their experiences to women with GDM from non‐CALD backgrounds.

**Materials and Methods:**

MEDLINE, EMBASE, PsycINFO, Scopus, WOS and CINAHL databases were searched for qualitative and quantitative studies which included data on the experiences of CALD background women with GDM during all stages of pregnancy. Quality appraisal utilized checklists for analytical cross‐sectional studies and qualitative research. Thematic analysis was performed using nVivo software.

**Results:**

Of the 3054 studies identified, 24 studies met the inclusion criteria. Data synthesis produced five key themes: (1) Response to diagnosis, (2) Experiences with self‐management, (3) Interactions with the healthcare system, (4) Mental health challenges and (5) Facilitators and barriers to support. Women with GDM from CALD and non‐CALD backgrounds similarly expressed mental health challenges, feeling burdened by recommendations, and challenges interacting with healthcare professionals (HCP). The major difference in experience was the cultural relevance of recommendations, especially related to diet recommendations.

**Conclusion:**

Gestational diabetes mellitus is a burdensome diagnosis for CALD and non‐CALD women, with CALD women uniquely experiencing a lack of culturally relevant recommendations for self‐management. The similarities and differences in experience call for optimisation of GDM management and support for women with GDM.

## Graphical abstract

Gestational diabetes mellitus (GDM) is a burdensome diagnosis for culturally and linguistically diverse (CALD) and non‐CALD women, with CALD women uniquely experiencing a lack of culturally relevant recommendations regarding diet and exercise. The similarities and differences in experience call for optimisation of GDM management and support for women with GDM from CALD and non‐CALD backgrounds.

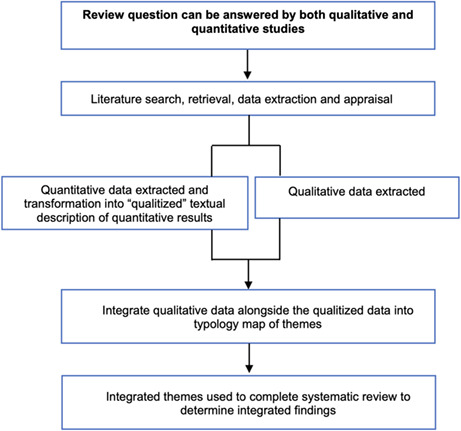

## 1 INTRODUCTION

Gestational diabetes mellitus (GDM) is most often diagnosed during the second or third trimester of pregnancy and defines new onset glucose intolerance in pregnancy.^1^ Women with higher glucose levels in pregnancy have an increased risk of foetal overgrowth (large‐for‐gestational age and macrosomia), caesarean delivery, neonatal hypoglycaemia, preterm delivery, delivery complications (shoulder dystocia and birth injury) and pre‐eclampsia.^2^ In addition, women with GDM are more likely to develop type 2 diabetes mellitus (T2D) after pregnancy.^2^, ^3^, ^4^ There are a range of known risk factors for GDM including a family history of T2D, maternal age > 40 years, pre‐pregnancy body mass index (BMI) > 30 kg/m^2^ and ethnicity.^3^

The prevalence of GDM impacts 12.8% of pregnancies globally,^5^ and in 2016–2017, the incidence of GDM in Australia reached a peak of 15%.^6^ Women from culturally and linguistically diverse (CALD) backgrounds have a preponderance for GDM diagnosis.^5^, ^6^, ^7^ Specifically, when compared to women born in Australia, the incidence of GDM was 1.6–2 times more likely in women born in India, South‐East Asia, North‐East Asia, Central Asia, North Africa and the Middle East.^6^, ^8^ Given the high rate at which GDM affects Australian women from CALD backgrounds, there is an unmet need to increase understanding of their experience.

Various studies have shown that a new diagnosis of GDM is associated with higher levels of stress and confusion,^9^, ^10^, ^11^ in addition to the already high levels of anxiety experienced by some women during pregnancy.^12^, ^13^ The uncertainty experienced at GDM diagnosis may be due to a lack of prior knowledge about GDM, in combination with bombardment of new information about GDM and its management.^9^, ^14^, ^15^ Women from CALD backgrounds have other stressors that may not be encountered by non‐CALD women, including language barriers requiring an interpreter, variations in health literacy levels, access to appropriate healthcare professionals (HCP), and varying cultural beliefs surrounding food, exercise and pregnancy.^16^, ^17^, ^18^, ^19^ In addition, women from a CALD background were more likely to experience antenatal and postnatal depression compared to women from non‐CALD backgrounds.^20^

The aim of this systematic review was to describe the experiences of women with GDM from CALD versus non‐CALD backgrounds, drawing on similarities and differences. Hence, we utilized a mixed methods approach to integrate qualitative and quantitative studies, to synthesize the experiences of women with GDM.

## 2 MATERIALS AND METHODS

This project was pre‐registered and approved in PROSPERO (CRD42020148779) and was conducted in accordance with a predefined PRISMA checklist^21^ (Appendix S1, S2). The data collection process included literature screening for primary studies published in peer‐reviewed journals. The inclusion criteria required a clear definition of GDM, inclusive of women with a new diagnosis of GDM or history of GDM, studies which included a defined group of women from CALD backgrounds separate from non‐CALD backgrounds, and included qualitative or quantitative data on the perspective or experience of GDM during any stage of pregnancy. Exclusion criteria for this systematic review were studies involving women with pre‐existing diabetes mellitus prior to pregnancy, no differentiation between GDM and T2DM, studies that did not separate data for CALD versus non‐CALD women's experiences, postpartum experience, or conference abstracts/proceedings.

The search strategy was developed using subject headings and key words. The initial search was performed in MEDLINE and adapted for each of the databases. The databases included MEDLINE, EMBASE, PsycINFO, Scopus, WOS and CINAHL, searched from inception to February 2022. The key terms were developed around the two areas: (i) GDM, and (ii) culturally and linguistically diverse background women. Other key concepts including experience, non‐CALD comparison and study design were not included because they could not be formulated into an inclusive search strategy (Appendix S3). Though study inclusion was not restricted by country of publication, the systematic review mandated written text in the English language.

### 2.1 Selection process and data extraction

Software packages, Endnote (version 20) and Covidence, were used to remove duplicate articles. Two reviewers, CH and RL, independently screened the titles, abstracts and full texts. Conflicts were settled via discussion. CH conducted the quality appraisal. Data extraction was completed in Covidence. An extraction template was created by CH, and two reviewers, CH and RL, completed the extraction. The main findings, themes, source of data, quotes and outcome measures were extracted. Additionally, study characteristics such as title, author, year, location, aim, study design, recruitment method and data analysis process were extracted.

### 2.2 Data analysis

**FIGURE 1:**
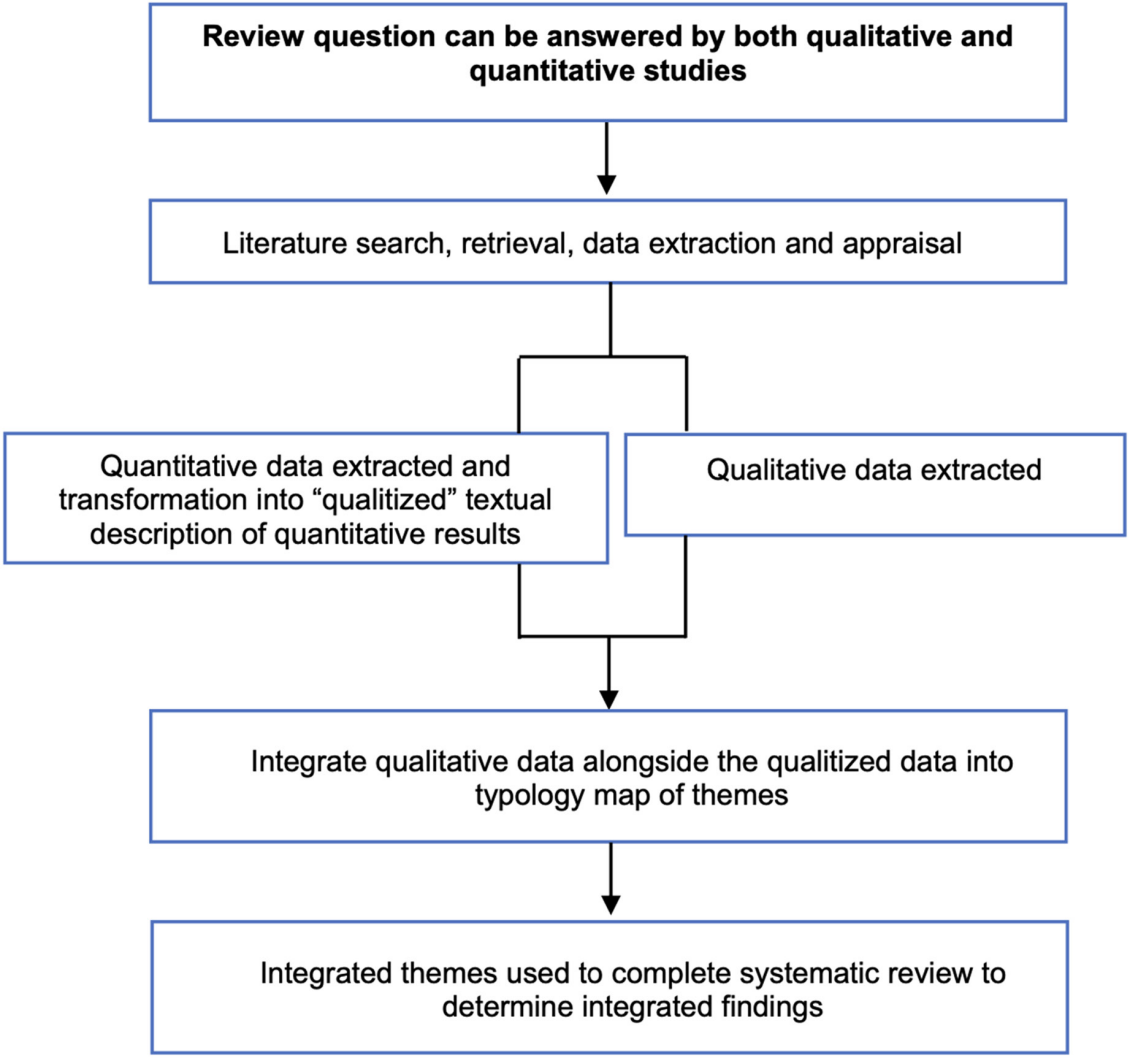
Convergent integrated approach to mixed methods systematic review.

Data analysis was guided by the Joanna Briggs Institute (JBI) recommendation for mixed methods systematic reviews.^22^ The review implemented a convergent integrated approach involving extraction, transformation, and integration of the qualitative and quantitative data. Given the heterogeneity of the quantitative data, the recommended data transformation method by JBI involved ‘qualitizing’ the quantitative data into qualitative data, facilitating a mutually compatible format of narrative description. In addition, all qualitative data were extracted as narrative description (Figure 1).

Once all quantitative data were transformed into narrative description and all qualitative data were extracted, the data were exported into nVivo (released in March 2020) for thematic synthesis. Thematic synthesis was guided by the process described by Thomas and Harden,^23^ involving extraction of text and quotes coded verbatim, line‐by‐line, then relationships between codes were developed into sub‐themes. Then, analytical themes were developed to interpret the findings.^23^ A typology map was developed of the relevant analytical themes, sub‐themes and descriptive themes from quotes. Table 1 outlines the typology map with relevant themes labelled respectively as third‐order, second‐order and first‐order themes.

**TABLE 1:** Typology of experiences of gestational diabetes in culturally and linguistically diverse background (CALD) and non‐CALD background.

Third‐order themes	Second‐order themes	CALD: first‐order themes	Non‐CALD: first‐order themes
Response to diagnosis	Emotional response Making sense of GDM	Women were confused, worried and sad about diagnosis Disbelief about diagnosis of GDM and the seriousness of it	Women were panicked and frightened about diagnosis Mild disbelief about diagnosis and the seriousness of it
Experiences with self‐management	Comprehension of recommendations Compliance with recommendations Cultural relevance of recommendations Burden of recommendations	Poor knowledge of GDM and difficulties comprehending the requirements for dietary modifications, exercise and glucose monitoring Motivation to follow recommendations was attributes to the baby's well‐being Struggles were associated with the restrictive nature of recommendations Dissatisfaction with the ones sized fits all approach to dietary recommendations Restrictive diet and blood glucose monitoring are cumbersome and energy consuming Separate dinners required for other family members	Average knowledge of GDM, clear comprehension of dietary requirements and glucose monitoring Motivation was intrinsic and in the interest of the baby's well‐being Struggles associated with finding time and small serving sizes Burden expressed for dietary requirements, glucose monitoring demands and finding time for exercise around family commitments. Loss of own time to GDM management
Interaction with healthcare system	Access to care Support from health professionals	Overwhelmed by the number of appointments required Difficult to find convenient time for appointment Inhibited by limited time for consultations Limited cultural competency of HCP	Difficult to organize appointments Difficult to have at convenient time Supportive diabetes educators Frustration towards wait times and lack of consistent information
Mental health challenges	Concerns for baby Anxiety, guilt and loss of control Importance of well‐being	Worries for the health of the baby Concerns about developing diabetes after pregnancy Blaming themselves for diagnosis Lack of ownership over pregnancy Good health perceived as resilience, lack of stress, feeling healthy and able to do everything	Worries for the health of the baby Guilt and self‐blame associated with GDM diagnosis Fear of trying different dietary options in case it makes GDM worse Improving health as positive outcome
Facilitators and barriers to support	Emotional and family support Financial barriers Social and cultural challenges	Mothers and partners were key players in support More expense associated with GDM diet Difficulties with dining out and GDM diet at festivities	Partner as key support person Suggestion for peer GDM support groups

**FIGURE 2:**
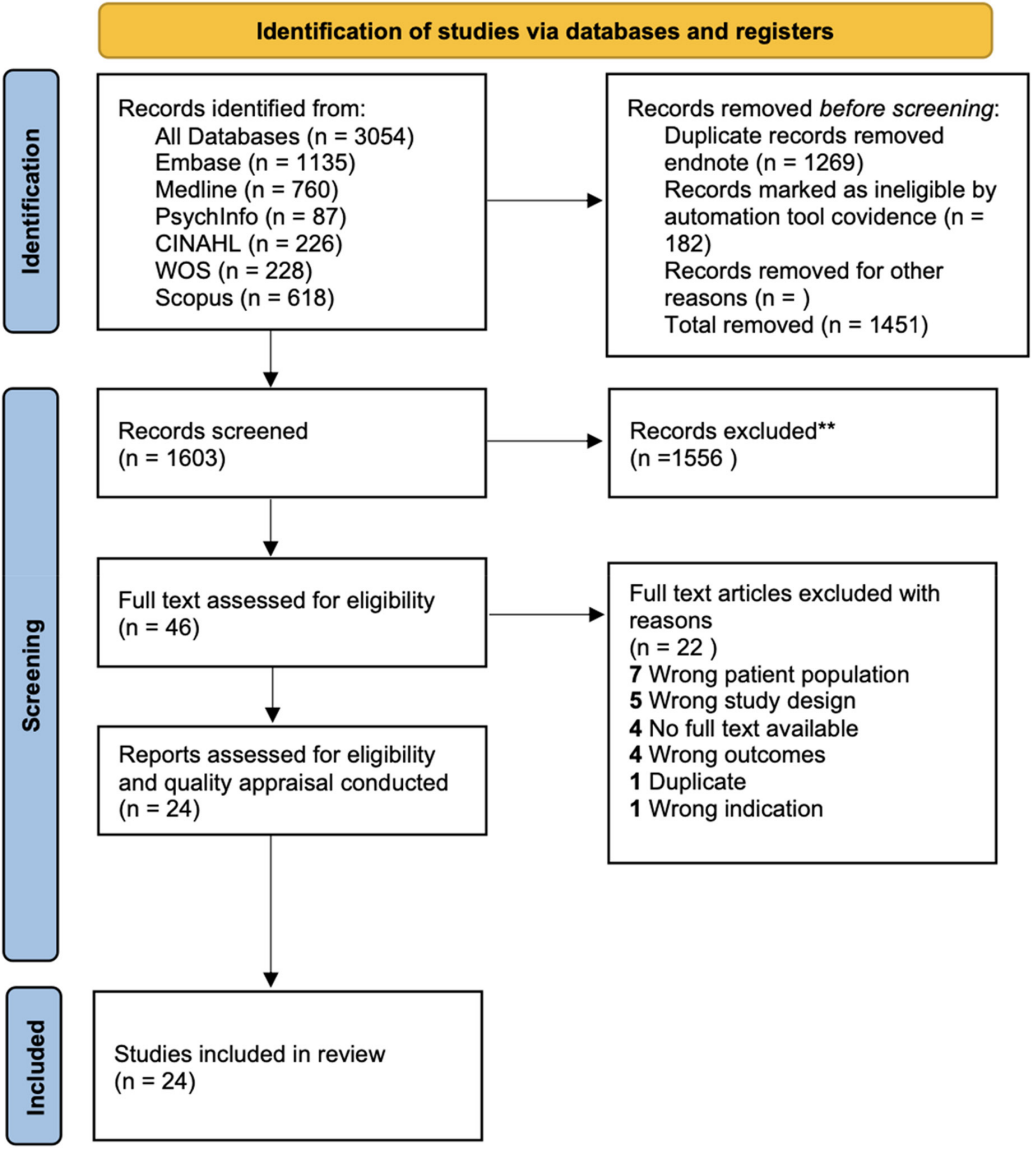
PRISM flow diagram.

Quality assessment was performed by using the JBI critical appraisal checklists for analytical cross‐sectional studies (Appendix S4).^24^, ^25^ The risk of bias was identified as low, moderate or high. Studies with a low risk of bias were of high quality and filled ‘yes’ for the majority of the JBI criteria, whereas studies of high risk of bias filled ‘no’ or ‘unclear’ for the majority of the JBI criteria (Figure 2).

## 3 RESULTS

The search strategy identified 3054 studies of which 1451 were removed via de‐duplication (Figure 2). Title and abstract screening excluded a further 1556 studies, leaving 46 studies for full text screening of which 22 were excluded. Data were extracted for 24 studies that met the eligibility criteria and were included in this systematic review.^11^, ^18^, ^19^, ^26^, ^27^, ^28^, ^29^, ^30^, ^31^, ^32^, ^33^, ^34^, ^35^, ^36^, ^37^, ^38^, ^39^, ^40^, ^41^, ^42^, ^43^, ^44^, ^45^, ^46^

Table 2 includes the characteristics of included studies. Sample sizes ranged from 9 to 898 women. Seventeen of the 20 qualitative studies used 1:1 semi‐structured interviews,^11^, ^26^, ^27^, ^28^, ^31^, ^32^, ^33^, ^34^, ^35^, ^36^, ^37^, ^38^, ^39^, ^40^, ^44^, ^45^, ^46^ two used focus groups,^41^, ^43^ and one used both^42^ as the main sampling methods for data collection. Data within the quantitative studies were extracted from questionnaires^18^, ^19^, ^29^, ^30^ and one from medical records.^29^ The studies were conducted across a range of countries including Australia (8), Sweden (6), Canada (3), Denmark (2), Norway (2), the United States (2) and the UK (1). A comparator to non‐CALD women was included in 7 of the 21 qualitative papers and 3 of the 4 quantitative papers.

**TABLE 2:** Characteristics of included studies.

References/Country	Title	Aim of study	Sample size (*n*)	Method of data collection/sampling context	Population details: current or previous GDM/method of recruitment/CALD background
*Qualitative studies*					
Bagger et al.^26^/Denmark	Perceptions of risk and motivation for healthy living among immigrants from non‐western countries with prior gestational diabetes mellitus living in Denmark	To explore perceptions of risk and motivation among immigrant women from non‐western countries with prior GDM living in Denmark	CALD = 12/non‐CALD = 0	Semi‐structured interview	Previous GDM diagnosis/clinic patients/East Asia (China/Korea/Japan); South East Asia (Filipino/Vietnamese); South Asia (Indian subcontinent); Central Asia (Middle East)
Bandyopadhyay^27^/Australia	Gestational diabetes mellitus: a qualitative study of lived experiences of South Asian immigrant women and perspectives of their healthcare providers in Melbourne, Australia	To gain a better understanding of the lived experiences of South Asian women and their experiences of self‐management and their healthcare providers, perspectives of treatment strategies	CALD = 23/non‐CALD = 0	Semi‐structured interview	Current GDM/clinic patients/South Asia (Indian subcontinent)
Bandyopadhyayet al.^28^/Australia	Lived experience of gestational diabetes mellitus among immigrant South Asian women in Australia	To explore the experiences and understandings of South Asian women in Melbourne, Australia, after diagnosis with GDM	CALD = 17/non‐CALD = 0	Semi‐structured interview	Current GDM/clinic patients/South Asia (Indian subcontinent)
Carolan et al.^31^/Australia	Women's experiences of factors that facilitate or inhibit gestational diabetes self‐management	To explore the factors that facilitated or inhibited gestational diabetes self‐management among women in a socially deprived area	CALD = 10/non‐CALD = 5	Semi‐structured interview	Current GDM/clinic patients/East Asia (China/Korea/Japan); South East Asia (Filipino/Vietnamese); South Asia (Indian subcontinent); Caucasian; Other: Arabic
Carolan‐Olah et al.^11^/United States	The experience of gestational diabetes mellitus (GDM) among Hispanic women in a U.S. border region	To explore the experiences of Hispanic women of Mexican origin with gestational diabetes mellitus (GDM)	CALD = 18/non‐CALD = 0	Semi‐structured interview	Current GDM/clinic patients/hispanic
Dayyani et al.^32^/Denmark	A qualitative study about the experiences of ethnic minority pregnant women with gestational diabetes	To explore how non‐Western ethnic minority pregnant women experience the hospital‐based information about gestational diabetes mellitus and how they integrate this information into their everyday life To investigate how health literacy and distributed healthy literacy affect this process	CALD = undefined/non‐CALD = 0	Semi‐structured interview	Current GDM/clinic patients/Central Asia (Middle East); Western Asia (Eastern European); Black; Other: Morocco
de Sequeira et al.^33^/Canada	Culturally tailored resources for South Asian immigrant women with gestational diabetes: do they work and what's missing? a qualitative study	To explore, among South Asian immigrant women with GDM in Toronto and the greater Toronto Area: (1) awareness of diabetes education and management resources (i.e. pamphlets from community fairs, Internet‐based resources, recipe cards and educational material for managing diabetes); (2) trust of information and recommendations from different resources and varying healthcare professionals (e.g. nurse vs physician); and (3) barriers and facilitators for diabetes management resources	CALD = 13/non‐CALD = 0	Semi‐structured interview	Current GDM/clinic patients/South Asia (Indian subcontinent)
Hjelm et al.^34^/Sweden	Swedish and Middle Eastern‐born women's beliefs about gestational diabetes	To compare beliefs about health and illness between women born in Sweden and the Middle East who developed gestational diabetes (GD)	CALD = 14/non‐CALD = 13	Semi‐structured interview	Current GDM; previous GDM diagnosis/clinic patients/Central Asia (Middle East)
Hjelm et al.^35^/Sweden	Gestational diabetes: prospective interview‐study of the developing beliefs about health, illness, and health care in migrant women	To explore the development over time of beliefs about health, illness, and health care in migrant women with GDM born in the Middle East living in Sweden and to study the influence on self‐care and care seeking	CALD = 14/non‐CALD = 0	Semi‐structured interview	Current GDM; previous GDM diagnosis/clinic patients/Central Asia (Middle East)
Hjelm et al.^36^/Sweden	A qualitative study of developing beliefs about health, illness and healthcare in migrant African women with gestational diabetes living in Sweden	To explore the development over time, during and after pregnancy, of beliefs about health, illness and healthcare in migrant women with GDM born in Africa living in Sweden, and to study the influence on health‐related behaviour, including self‐care and care seeking	CALD = 9/non‐CALD = 0	Semi‐structured interview	Current GDM/clinic patients/other: African
Hjelm et al.^37^/Sweden	Gestational diabetes: changed health beliefs in migrant women from five Asian countries living in Sweden: a prospective qualitative study	To explore the temporal development of beliefs about health, illness and health care in migrant women with gestational diabetes (GD) born in Asia residing in Sweden, and the influence on health‐related behaviour in terms of self‐care and seeking care	CALD = 9/non‐CALD = 0	Semi‐structured interview	Current GDM/clinic patients/East Asia (China/Korea/Japan); South East Asia (Filipino/Vietnamese); South Asia (Indian subcontinent)
Hjelm et al.^38^/Sweden	Management of gestational diabetes from the patient's perspective—a comparison of Swedish and Middle Eastern born women	To explore patients‚ evaluation of a specialized diabetes clinic for management of women with gestational diabetes born in Sweden and the Middle East and its contribution to a decreased level of stress and improved coping capability to promote health in patients receiving care	CALD = 14/non‐CALD = 13	Semi‐structured interview	Current GDM/clinic patients/Central Asia (Middle East)
Hjelm et al.^39^/Sweden	Beliefs about health and illness in Swedish and African‐born women with gestational diabetes living in Sweden	To explore beliefs about health and illness in women with gestational diabetes born in Sweden and Africa living in Sweden. A further aim is to study the influence of beliefs on self‐care and care seeking	CALD = 10/non‐CALD = 13	Semi‐structured interview	Current GDM/clinic patients/Caucasian; other: African
Jirojwong et al.^40^/Australia	Going up, going down: the experience, control and management of gestational diabetes mellitus among Southeast Asian migrant women living in urban Australia	To explore how Southeast Asian migrant women in Australia experience and manage GDM	CALD = 19/non‐CALD = 0	Semi‐structured interview	Current GDM/clinic patients/South East Asia (Filipino/Vietnamese)
Oza‐Frank et al.^41^/United States	Healthcare experiences of low‐income women with gestational diabetes and suggestions for improving care	To describe the healthcare experiences of a diverse sample of low‐income women with a history of GDM, including suggestions for improving care	CALD = 19/non‐CALD = 0	Focus group discussions	Current GDM; previous GDM diagnosis/clinic patients/African American; Hispanic; Appalachian
Parsons et al.^42^/UK	Experiences of gestational diabetes and gestational diabetes care: a focus group and interview study	To explore the experiences of GDM and GDM care for a group of women attending a large diabetes pregnancy unit in southeast London, UK, to improve care	CALD = 37/non‐CALD = 13	Semi‐structured interview and focus group discussions	Current GDM; previous GDM diagnosis; other: GDM in the last 5 years/clinic patients/East Asia (China/Korea/Japan); South East Asia (Filipino/Vietnamese); South Asia (Indian subcontinent); Central Asia (Middle East); Caucasian; Black
Sharma et al.^43^/Norway	Understanding mechanisms behind unwanted health behaviours in Nordic and South Asian women and how they affect their gestational diabetes follow‐up: a qualitative study	To advance the knowledge regarding the mechanisms behind suboptimal follow‐up in the Nordic and South Asian women with previous GDM by comparing (1) their experiences, (2) health and disease perceptions and (3) barriers to and facilitators of health‐promoting behaviours	CALD = 18/non‐CALD = 10	Focus group discussions	Current GDM/clinic patients/South Asia (Indian subcontinent); Caucasian
Siad et al.^44^/Canada	Understanding the experiences of East African immigrant women with gestational diabetes mellitus	To document the impact of a diagnosis of GDM and the perceptions of diabetes care among EA immigrant women	CALD = 10/non‐CALD = 0	Semi‐structured interview	Current GDM/clinic patients/other: East Africa
Wah et al.^45^/Australia	Self‐management of gestational diabetes among Chinese migrants: a qualitative study	To explore the understanding and self‐management experiences of GDM among Chinese migrants	CALD = 18/non‐CALD = 0	Semi‐structured interview	Current GDM/clinic patients/East Asia (China/Korea/Japan)
Wan et al.^46^/Australia	Ethnic differences in dietary management of gestational diabetes mellitus: a mixed methods study comparing Ethnic Chinese Immigrants and Australian women	To explore the perceptions and experiences of dietary self‐management among ethnic Chinese migrants with GDM (herein referred to as Chinese participants) compared with those of Australian‐born white women with GDM (herein referred as white participants) To examine ethnic differences in nutritional intake and patterns of supplement use	CALD = 42/non‐CALD = 30	Semi‐structured interview	Current GDM/clinic patients/East Asia (China/Korea/Japan; Caucasian)
*Quantitative studies*					
Banerjee et al.^29^/Canada	Reported health behaviour changes after a diagnosis of gestational diabetes mellitus among ethnic minority women living in Canada	To examine differences in self‐reported health behaviour changes during pregnancy between ethnic minority and Caucasian women diagnosed with GDM in Ontario, Canada	CALD = 539/non‐CALD = 359	Questionnaire (Likert scale); semi‐structured interview; other: medical charts	Current GDM/clinic patients; other: paper, telephone or online questionnaire/East Asia (China/Korea/Japan); South East Asia (Filipino/Vietnamese); South Asia (Indian subcontinent); Central Asia (Middle East); Latin American; West Indian; First Nations/Aboriginal; Caucasian; Black; Pacific Islander; other: Filipino
Borgen et al.^30^/Norway	Knowledge of gestational diabetes mellitus at first consultation in a multi‐ethnic pregnant population in the Oslo region, Norway—a cross‐sectional study	To investigate knowledge of GDM among a multi‐ethnic pregnant population at first consultation for GDM in the Oslo region in Norway	CALD = 130/non‐CALD = 108	Questionnaire (yes/no); Questionnaire (Likert scale)	Current GDM/clinic patients; voluntary/East Asia (China/Korea/Japan); South East Asia (Filipino/Vietnamese); South Asia (Indian subcontinent); Western Asia (Eastern European); other: African
Carolan et al.^18^/Australia	Attitudes towards gestational diabetes among a multi‐ethnic cohort in Australia	To examine the attitudes and beliefs towards gestational diabetes of a multi‐ethnic sample of pregnant women with gestational diabetes	CALD = 78/non‐CALD = 62	Questionnaire (Likert scale)	Current GDM/clinic patients/South East Asia (Filipino/Vietnamese); South Asia (Indian subcontinent); Caucasian
Carolan et al.^19^/Australia	Knowledge of gestational diabetes among a multi‐ethnic cohort in Australia	To explore knowledge about gestational diabetes (GDM) among a multi‐ethnic sample of women who were receiving antenatal care in Melbourne, Australia	CALD = 78/non‐CALD = 62	Questionnaire (yes/no); Questionnaire (pictorial); Questionnaire (Likert scale)	Current GDM/Clinic patients/South East Asia (Filipino/Vietnamese); South Asia (Indian subcontinent); Caucasian

Abbreviations: CALD, culturally and linguistically diverse background; GDM, gestational diabetes mellitus.

### 3.1 Quality appraisal

All studies included were assessed to be of high quality, with a low risk of bias. All studies included a study aim and method of sampling, though they did not always state the method of GDM diagnosis or acknowledge the relationship between researcher and participant.

### 3.2 Thematic analysis and data synthesis

Five key themes were developed from the synthesis of qualitative and quantitative data, presented in Table 1. The themes included (1) Response to diagnosis, (2) Experiences with self‐management, (3) Interactions with the healthcare system, (4) Mental health challenges, and (5) Facilitators and barriers to support.

The qualitative and quantitative results were narratively synthesized together according to themes below. Details of the relevant quotations and findings to support each theme from both the qualitative and quantitative studies are summarized below (Tables 3, 4, respectively).

**TABLE 3:** Summary of qualitative findings and supporting quotations from participants.

Theme	Sub‐theme	CALD	Non‐CALD
Response to diagnosis	Emotional response^11^, ^27^, ^28^, ^31^, ^32^, ^34^, ^36^, ^39^, ^42^, ^43^, ^44^	‘Upset’, ‘shocked’, ‘disappointed’, ‘scared’, ‘fearful’, ‘felt like crying’, ‘disbelief’, ‘surprised’^28^ ‘…I was crying … This is serious… I have GDM’^32^ ‘I freaked out and … I was shocked I was like I don’t know why or what was it … so I was kind of lost, scared, I didn’t know what to do’^11^ ‘I felt worried and sad, I went home to cry’^36^ ‘I think I was so overwhelmed I just wanted to get out of there’^42^	‘I panicked…I was terribly frightened’^34^ ‘Being in panic’^39^ ‘No time to think it through’^31^
	Making sense of GDM^11^, ^26^, ^31^, ^32^, ^34^, ^35^, ^36^, ^37^, ^38^, ^39^, ^42^, ^43^, ^46^	‘I didn’t know how serious this disease was…I didn’t perceive it as particularly serious’^36^ ‘It is not so serious and it will probably disappear after delivery’^39^ ‘At first it was difficult to accept that I had diabetes…I was a little scared.’^11^ ‘I believe I don't have [diabetes], I don't know, but I'm not diabetic’^42^ ‘I didn’t understand very clearly … It was quite difficult to get my head around it’^42^ ‘I don’t think I understand it in‐detailed. I think I have a basic understanding’^46^	‘I didn’t really want to know whether I had diabetes… it is easy to think that maybe I am done with it’^43^
Experiences with self‐management	Comprehension of recommendations^11^, ^28^, ^31^, ^32^, ^35^, ^36^, ^37^, ^39^, ^40^, ^45^, ^46^	*Diet* ‘Your diet is restricted…then the baby does not get proper nutrition to grow’^28^ ‘I felt hungry, always. But I am not allowed to eat anything’^46^ ‘I think you can eat and drink whatever…just eat in small portions’^45^ ‘You can eat maybe two slices of pizza but with a salad. You can eat…If your sugar levels are good…a piece of cake but that's it for the day’^11^ *Exercise* ‘I've heard it's good to walk.’^35^ ‘If the blood sugar is normal then I wouldn’t do exercise’^45^ ‘I had to start doing exercise every third day’^11^ ‘They told me to walk … I walked regularly every day half an hour to 1 h’^40^ *Blood glucose* ‘…four or five… or I don’t know if it is six or more than six but I have heard nine sometimes’^39^ ‘They just told me, ‘You've got diabetes, you'd better control…’ But they didn’t tell me… like why it's really important to monitor your sugar levels…’^31^ ‘I'm confused, I don’t understand …my sugar after 1 h is often right on or above 7.5, but after 2 h, it could go below 6’^45^	*Diet* ‘Half of the plate of salad or green vegetables… and I have a bit of carb, a quarter of the plate, whatever the serving size like the sweet potato… and then like the rest protein.’^46^ ‘The type of carb like I will have the brown bread instead of white bread…Basically I will get all the low GI options’^46^ ‘I will generally snack straight away. So just I don’t starving by the time lunch or dinner comes around’^46^ *Blood glucose* ‘The group session that we had… enough understanding and tools that we needed… enough knowledge …to manage my sugar’^46^ ‘it's the pancreas that doesn’t work fully. The load on the body increases while pregnant…it can’t handle all the sugar…it increases’^35^
	Compliance with recommendations^11^, ^26^, ^27^, ^28^, ^31^, ^32^, ^34^, ^35^, ^36^, ^39^, ^40^, ^41^, ^45^, ^46^	*Struggles* ‘I hadn’t really looked after what I had been eating…I really wasn't trying’^31^ ‘I restrict my diet…The portion is making me really frustrated’^46^ ‘They suggested insulin after that first week and I didn’t want’^31^ ‘I am still very against the idea of using insulin’^45^ ‘…the impact of this pregnancy on the body, it's hard to walk’^45^ ‘It's really hard just to stick to’^11^ ‘I don’t feel full. I have to eat a lot and then after half an hour when I measure the BG will be very high and that is a challenge‘^32^ *Motivation* ‘It will affect your baby, so you have to do the right thing by the baby’^31^ ‘Your only thought is for the baby and for a favourable pregnancy outcome’^28^ ‘I fight for my baby's sake… I don’t care so much about myself’^37^ ‘I will do what the doctor ask me to do. …follow what the doctor suggested me to eat’^46^ ‘I listen to the doctor's advice …eat what the doctor has advised’^40^	*Struggles* ‘Like where do you find the time… having to actually think about it, prepare it and cook healthy food…That's my biggest change, probably, going from never cooking’^31^ ‘I feel like sometimes I was having much smaller servings of things and I am just not having enough’^46^ *Motivation* ‘I was very determined to make sure I could do absolutely anything within my power to not allow any, something to happen to the baby. I knew I had brought it on myself by being overweight. I felt very responsible’^31^ ‘to live a healthy life with healthy food and exercise’^34^ ‘I realized it was up to me…no one else… there was no point in cheating… I would be just cheating myself’^31^
	Cultural relevance of recommendations^27^, ^28^, ^31^, ^32^, ^33^, ^41^, ^42^, ^43^, ^44^, ^45^, ^46^, ^49^	*Diet* ‘If the suggestions could be more relevant to Chinese lifestyle, a lot of things that have been taught currently are not meaningful.’^46^ ‘It is all Canadian …So, then I have to change it to my things. I think if country‐specific patterns, sample diets [were created], that could be better.’^33^ ‘We are told is that all this “English” food is good and ours is not…people here eat steamed vegetables and meat … But we are used to eating different kinds of things’^27^ ‘Currently everything is a matter of trial and error for us… If they can provide a bit more customized diet plan for us, …like specially the roti … what should be the replacement’^27^ ‘[health professionals] need to know what our cultural food is for them to be able to help us more in the future’^44^ ‘It's [recommended dietary plan] closer to the Cantonese eating habit… I'm from the Northern areas (of China), so I eat kind of differently’^45^ *Exercise* ‘In my family, there is a shared opinion about exercising in front of boys’^43^ ‘She also suggests me to walk at least 30 min per day, but I am not doing, … here it is so cold for me, I am afraid to get outside’^32^	
	Burden of recommendations^11^, ^26^, ^31^, ^32^, ^35^, ^36^, ^42^, ^43^, ^44^, ^46^	‘A test you're potentially failing four times a day’^42^ ‘I had to measure [my blood glucose] before and after every main meal. That's six times a day. And you know, that is a lot. I find it very cumbersome and energy consuming’^26^ ‘The saddest part, having to check, yeah, four times a day, it was just hard’^42^ I have got to work, I think checking two or three times a day is already the best I can do’^45^ ‘Moreover, if they wake up and I have gone out to exercise, then it will be a complete chaos’^43^ ‘I must live in a routine now, right. My eating has to be on schedule, and testing and all of that. So it makes it stressful’^44^	‘Because I do shift work, I've found I've had to make a strict monitoring sort of system. So, I set alarms on my phone every time I have something to eat, so I remember to do the 2 h afterwards [BGL], otherwise I'll just forget’^31^ ‘The doctor said to walk for an hour after meals. I mean, I start (work) at seven and finish at three and then I've got to pick my daughter up from school. Trying to fit that in, it's just … I think, well, God, I'll be dead by the time I get back, you know?’^31^ ‘We had three different dinners every night… I could have eaten the same thing without the carbs… but so boring’^31^ ‘you are not free, you need to control diet, time for meals…monitoring blood glucose…’^35^
Interaction with healthcare system	Access to care^28^, ^31^, ^32^, ^35^, ^36^, ^37^, ^38^, ^40^, ^41^	‘I have to actually go back there [clinic] twice a week, I think the dietitians you can only book them on a Friday…but the obstetrician, only …on a Monday… so that is hard with work’^31^ ‘It's difficult…it takes a long time before I get an appointment and then I don’t know when I get the time whether it might be difficult to get here…’^35^ ‘It is difficult to book a time (at the physician)’^37^	‘It has been difficult to get a line on the telephone…you have to phone between 8:30 and 9:00 and if you then have a question that arises at 12 o'clock you get a message that you have to wait until next day.’^38^ ‘Accessibility in general during working hours, because I have to work from 7 to 2…if there are five diabetes specialist nurses somebody ought to be able to lift the receiver…it's not likely that everybody should have it at the same time’^38^
	Support from health professionals^26^, ^31^, ^32^, ^33^, ^35^, ^36^, ^38^, ^40^, ^41^, ^42^, ^43^, ^44^, ^45^	*Positive* ‘The dietitian is very, very kind and so together with her we found also in the Internet some products; try this and this and this’^32^ ‘I was shocked how much I learned from the dietitian… it was just amazing’^41^ *Negative* ‘They just had a short time so there was no chance to tell how you feel’^36^ ‘I want them to treat me like a human. Treat me, not my diagnosis’^44^ ‘They make it as if you don’t care about your health or your baby’^44^ ‘I am associated with the dietitian… but there is a slight gap here. So I try to convert everything from my side to present it to her.’^33^ ‘[The doctors] flat out tell you you’re going kill your child. So, I was very careful with what I told him but that made me feel uneasy’^41^ ‘So, we’re already stressed. And now you’re going to shout at us for not bringing the monitor and having a KFC…it just feels like you’re getting in trouble’^42^ ‘I often got the feeling that health professionals thought that I just hadn't been thinking about my health or taking care of myself or putting any effort and thought into it’^42^	*Positive* ‘The diabetic clinic, they clearly were very supportive’^42^ ‘I had a lady (educator) that was really excellent…She would listen to my concerns and we had a conversation rather than just a one‐way flow of information’^31^ *Negative* ‘You get a list of food but no instructions… you don’t know “if I eat a smaller potato is that okay?” And then you follow it (the diet) through the pregnancy.… “Follow this diet, you'll be fine, off you go”’^31^ ‘The midwife does not know all about diabetes and those who work with diabetes don’t know all about the baby’^38^ ‘It would have been good if the midwife had been able to tell me more when I was there…you should not need to wait for 2 weeks before you can meet a dietitian’^38^
Mental health challenges	Concerns for baby^31^, ^35^, ^37^, ^42^, ^46^	‘It creates a lot of anxiety…I don’t want to eat now because I don’t know if I harm the baby. But I feel hungry’^46^ ‘First of all I am very worried for the baby …that he will be affected by the diabetes.’^35^ ‘For me it doesn’t matter if I have diabetes, but I don’t want my child to have diabetes in the future.’^35^ ‘All I could think about was how I'd damaged my baby’^42^ ‘It's not just like a diet you know…because he's going to know if I cheat, my baby is going to know’^11^ ‘I was very worried, I felt sick…All I thought about was what could happen to the baby.’^37^ ‘I'm afraid, I'm afraid also for the future … and also for my child’^32^	‘I was very determined to make sure I could do absolutely anything within my power to not allow something to happen to the baby. I knew I had brought it on myself by being overweight…I felt very responsible’^31^ ‘worried for the child…that the child will be too big…if you get type 2 diabetes… that worries me’^35^
	Anxiety, guilt and loss of control^11^, ^26^, ^27^, ^32^, ^35^, ^38^, ^40^, ^42^, ^43^, ^46^	‘It was a really difficult period. I was really worried whether I would get diabetes afterwards.’^26^ ‘I feel like I can’t go out anywhere or just enjoy anything anymore. … all my concentration is on what I eat, walk, how much I eat, walk, and eat, walk, eat, walk, that's it, I don’t do anything else’^27^ ‘It [GDM] really did affect me and I think it's probably one of the worst times I've had in my life actually’^42^ ‘My husband sometimes says, “Have you noticed the [hospital's] behaviours, it's not your child it's their child!’^42^ ‘I've got gestational diabetes, you know, people are looking at me like oh, I'm overweight, that's why I've got it’^42^ ‘I wanted to have another baby and since I got this I do not want to anymore’^11^ ‘When I tested my blood sugar, if it was high it made me very anxious’^40^ ‘I have not been so worried that I have sought help from religion’^38^	‘If you’re fat, then you are dumb. You have been unable to make the right decisions in life’^43^ ‘I just too scared to try anything else…I have exactly the same food …I am too scared to test the theory’^46^
	Importance of well‐being^34^, ^35^, ^37^	‘if you have health then you are able to do everything’^34^ ‘Health means everything…I need to feel healthy… feel well… able to do everything’^37^ ‘If you feel that you have good health then the world becomes very good for you…If you have health then you can do everything’^35^	‘I was very grateful that the test was made …I felt I had the possibility of influencing my own well‐being as well as that of the child’^35^ ‘Avoiding stress, being occupied with something…having a job that you are pleased …rest and a good night's [sleep] …positive thoughts’^35^
Facilitators and barriers to support	Emotional and family support^31^, ^32^, ^33^, ^34^, ^38^, ^45^	‘…my mother… she usually calls every day (from India)… and she always try to give me some home recipes, so that I get through my diabetes’^31^ ‘My kids and my husband remind me to take my pills, check my blood sugar level every time.’^33^ ‘He'll [my husband] go and check online that what I'm having has the proper content of carbs in it… all these things that would have taken a lot of time for me, energy from me’^33^ ‘If it's not because of my husband, I couldn’t have made it this far… he would just remind me not to’^45^ ‘If I pray to God I think he will help me to get well’^34^ ‘When I see the other women with the same symptoms and problems, I felt relieved that there was somebody like me’^32^ ‘It would have been interesting…if there had been a group where you could meet other persons who had had gestational diabetes…very useful’^38^ ‘if you have those who are in similar situations as you to work with you and to help you, it's easier’^41^	‘Having that support base, having someone kind of do it with you… makes you feel you’re not alone doing it’^31^ ‘My husband was very supportive… he helped me get over things like the finger pricks… he did his own 1 day just to show it wasn't a big deal’^31^ ‘If had been a group where one could meet other people who had had gestational diabetes very useful, to meet others that could talk to us about this’^34^
	Financial barriers^26^, ^31^, ^32^, ^35^, ^41^, ^44^, ^46^	‘Health is more important than money.’^35^ ‘You can get a little loaf of the healthy bread like this for like $2.50. I can get a giant loaf …for like $2…It goes so much further, that little bit counts towards the end of the week’^41^ ‘It is very expensive being pregnant’^44^ ‘I don’t have health care…I have to pay every time I visit the doctor…with my money…$50 each visit’^44^	
	Social and cultural challenges^11^, ^31^, ^32^, ^34^, ^44^, ^45^	‘It is difficult for me if my friends invite me they make a lot of food and it is difficult to weigh my bread and figure out how much (to eat)’^32^ ‘They cower … When we told them [I have] diabetes, they [are] scared’^44^ ‘Says it's contagious’^44^ ‘I don’t know why people they make it secret. My friend she never tell me, but she told me when I told her’^44^ ‘I have to go out for family dinners … Everyone eats so you need to eat too, then you will easily eat too much’^45^ ‘Having dinner at other people's houses [is difficult]… you’re not in control of what's being served … when you visit’^11^ ‘The physician wanted to prevent me from fasting, but I have done it anyhow’^34^	

**TABLE 4:** Narrative description of quantitative findings and characteristics of measurement tool used.

	Measurement tool	Response to diagnosis		Experiences with self‐management		Interaction with healthcare system		Mental health challenges	
		CALD	Non‐CALD	CALD	Non‐CALD	CALD	Non‐CALD	CALD	Non‐CALD
Banerjee et al.^29^/Canada	Non‐standardized questionnaire: Based on the Proschaska's transtheoretical model of change: measured behaviour modifications and readiness for change Evaluated: via modified Delphi Panel approach			*Diet* 80.4% reduced their meal portion sizes following diagnosis of GDM 94.4% reduced their intake of sweets and junk foods *Exercise* 58.8% increased their physical activity levels following diagnosis	*Diet* 68.2% reduced their meal portion sizes following diagnosis of GDM 93.0% reduced their intake of sweets and junk foods *Exercise* 39.0% increased their physical activity levels following diagnosis				
Borgen et al.^30^/Norway	DAS3, modified questionnaire (based off^18^) Modifications sensitive to questions regarding GDM rather than T1/2DM Measured: correct answers = two points, correctly identified false answers = one point, and incorrect answers = 0 points	*Knowledge* 40% had a poor knowledge of GDM 50% had an average knowledge of GDM 10% had an excellent knowledge of GDM	*Knowledge* 6.5% had a poor knowledge of GDM 71.3% had an average knowledge of GDM 22.2% had an excellent knowledge of GDM						
Carolan et al.^18^/Australia	DAS3 modified questionnaire Modifications sensitive to questions regarding GDM rather than T1/2DM Measured using Likert scale	*Seriousness of GDM* CALD women rated the seriousness of GDM with a score of 20.28/30^a^ which was lower than non‐CALD women	*Seriousness of GDM* Non‐CALD women rated the seriousness of GDM with a score of 21.05/30	*Blood glucose* CALD women rated the value of tight blood glucose control as low with a score of 28.18/40^a^; however, the values varied between ethnic groups and level of education was believed to influence the variation in scores	*Blood glucose* non‐CALD women rated the value of tight blood glucose control as high with a score of 30.50/40	*Health professionals training* CALD women rated the need for specialized training for health professionals providing GDM care as high with a score of 24.31/30^a^	*Health professionals training* non‐CALD rated the need for specialized training for health professionals providing GDM care as high with a score of 24.15/30	*Psychological impact* CALD women rated the psychological impact of GDM to only be moderate with a score of 20.23/30	*Psychological impact* Non‐CALD women rated the psychological impact of GDM to only be moderate with a score of 21.07/30
Carolan et al.^19^/Australia	Diabetes Knowledge Scale, validated via Cronbach's alpha coefficient of 0.92 Additional seven questions on pregnancy and GDM Modifications sensitive to questions regarding GDM rather than T1/2DM	*Knowledge* 35.5%^a^ had excellent knowledge of GDM 33%^a^ had poor knowledge of GDM	*Knowledge* 54.8% had excellent knowledge of GDM 19.4% had poor knowledge of GDM	*Diet* 67.9%^a^ expressed the correct knowledge on carbohydrate food values 39.7%^a^ expressed the correct knowledge of carbohydrate food substitutions *Blood glucose* 78.3%^a^ knew the normal ranges for blood sugar levels	*Diet* 88.7% expressed the correct knowledge on carbohydrate food values 31.1% expressed the correct knowledge of carbohydrate food substitutions *Blood glucose* 82.3% knew the normal ranges for blood sugar levels				

Abbreviation: DAS‐3, diabetes attitude scale version 3.

^a^ Some measures were aggregated by mean for ease of comparison and interpretation. Theme 5, facilitators and barriers to support, was omitted from this table as there were no quantitative findings that aligned with this theme.

### 3.3 Response to diagnosis

#### 3.3.1 Emotional response

The qualitative studies showed that an initial emotional response to the diagnosis of GDM was consistent across both CALD and non‐CALD women. Women from both groups described feeling panicked, shocked, confused, fearful, sad and stressed when first diagnosed; ‘I felt worried and sad, I went home to cry’^36^ Both groups expressed disbelief about their diagnosis and questioned ‘why them’ or what they had done ‘wrong’ and felt time pressure to make sense of the diagnosis quickly; ‘no time to think it through’.^31^

#### 3.3.2 Making sense of GDM

Both the quantitative and qualitative studies showed that confusion was the predominate feeling that women experienced as they came to terms with the diagnosis. Questions regarding the health of their baby and risk of developing long‐term diabetes frequently arose, with one quantitative study showing that 40% of CALD women had a poorer knowledge of GDM compared to 6.5% of non‐CALD women having a poor knowledge of GDM (Table 4).^30^ Despite this, both groups ranked the seriousness of GDM as low,^30^ with one quantitative study showing that CALD women rated the seriousness of GDM with a score of 20.28/30; and similarly non‐CALD women rating the seriousness of GDM with a score of 21.05/30 (Table 4).^18^In a qualitative study, one CALD woman expressed, ‘In the beginning I did not know how serious this disease was…I did not perceive it as particularly serious’.^36^

### 3.4 Experiences with self‐management

#### 3.4.1 Comprehension of recommendations

Many CALD women had a basic understanding of GDM and had difficulties comprehending the requirements of self‐management. CALD women expressed concerns that a restricted diet would negatively affect the baby's growth. From one qualitative study, some women felt that they were ‘not allowed to eat anything’^46^ and misunderstood the dietary advice given. For example, one woman stated that she drank water to counterbalance her high blood glucose.^32^ This was also represented by the quantitative date which showed that 67.9% of CALD women expressed the correct knowledge on carbohydrate food values compared to 88.7% of non‐CALD women expressing the correct knowledge on carbohydrate food values (Table 4).^19^

Many CALD women felt that the need for blood glucose monitoring was not adequately explained to them and consistent information was limited; ‘They just told me, “You've got diabetes, you'd better control with this and that.” But they didn’t tell me, like, what are the side effects for having sugar levels up’,^31^ nor what the variation in blood glucose values meant. The qualitative studies indicated that women struggled to understand what was required to maintain a stable blood glucose reading. Overall, there was a consensus among CALD women that a steep learning curve was associated with the self‐management of GDM. This directly contrasted the experience of non‐CALD women who felt enough information was given to monitor BGLs; ‘The group session that we had …[gave me]… enough knowledge… to manage my sugar’.^46^

#### 3.4.2 Compliance with recommendations

Both the quantitative and qualitative studies showed that women from both groups expressed that they were ‘willing to do whatever they were required to do’ in the interest of the baby's health^31^; ‘It will affect your baby, so you have to do the right thing by the baby. I do have the temptation. But those times I control, and I eat well for my baby because I [will] be there for the baby’.^31^ One quantitative study showed that CALD women reduced their intake of sweets and junk foods by 94.4% and non‐CALD by 93.0% (Table 4).^29^CALD women also expressed that motivation to follow the lifestyle modifications was to avoid needing insulin therapy, ‘I am still very against the idea of using insulin’.^45^

Women from both groups expressed struggles with maintaining a GDM‐specific diet. Women felt that the smaller meals and less carbohydrate left them feeling hungry and unsatisfied, ‘I restrict my diet…The portion is making me really frustrated’.^46^ CALD women expressed that the fluctuation in sugar levels, despite the care with diet, was also frustrating ‘I do not feel full. I have to eat a lot and then after half an hour when I measure the BG will be very high and that is a challenge’.^32^

#### 3.4.3 Cultural relevance of recommendations

Culturally and linguistically diverse women felt little effort was made by HCPs to individualize dietary recommendations, and expressed in the qualitative studies that in some instances culturally relevant information was not available; ‘Currently everything is a matter of trial and error for us… If they can provide a bit more customized diet plan for us, …like specially the roti… what should be the replacement?’^27^ The sense that recommendations were based around a western diet, with little consideration of adequate substitutions was evident, ‘…if the suggestions could be more relevant to Chinese lifestyle, a lot of things that have been taught currently are not meaningful’.^46^ Some women felt that their traditional food was perceived as bad and an ‘us versus them’ attitude was apparent; We are told that all this ‘English’ food is good and ours is not…people here eat steamed vegetables and meat… But we are used to eating different kinds of things.^27^ In addition religious festivities and events were also described as being associated with ‘high calorie celebratory foods’^31^ which made it difficult for women to participate in.

#### 3.4.4 Burden of recommendations

Women from both groups felt that regular glucose monitoring was a burden, ‘I had to measure [my blood glucose] before and after every main meal. That's six times a day… that is a lot’.^26^ Women from both groups expressed that a stringent routine was required to meet recommendations; ‘I must live in a routine now, right. My eating has to be on schedule, and testing and all of that. So it makes it stressful’.^44^ Women expressed that their partners and children did not follow the same diet as them, catering for their family and themselves separately. ‘We had three different dinners every night… I could have eaten the same thing without the carbs… but so boring’.^31^ Further, the notion of ‘finding time for everything’ was not feasible within some family routines, ‘if they [my children] wake up and I have gone out to exercise, then it will be complete chaos’.^43^

### 3.5 Interactions with the healthcare system

#### 3.5.1 Access to care

Women from both groups expressed that they felt overwhelmed by the number of appointments required. This meant multiple trips to the hospital in 1 week; ‘Sometimes… I have to actually go back there [clinic] twice a week, I think the dietitians only, you can only book them on a Friday…*but the obstetrician, only* …*on a Monday*… so that is hard with work’.^31^Women from both groups expressed that not only were the appointments time consuming, but they were also difficult to book due to long waiting times or not being available at a convenient time; ‘It's difficult…it takes a long time before I get an appointment and then I do not know when I get the time whether it might be difficult to get here…’^35^

#### 3.5.2 Support from health professionals

The quantitative studies showed that women from both groups rated the importance of specialized training for HCP as high, with scores of 24.31/30 and 24.15/30 for CALD and non‐CALD respectively (Table 4)^18^While the qualitative studies showed that some women attributed gaps in their information to the limited time for consultations; ‘They just had a short time so there was no chance to tell how you feel’.^36^ Some women expressed that HCPs did not appreciate the effort they were putting in; ‘I often got the feeling that health professionals thought that I just hadn't been thinking about my health or taking care of myself or putting any effort and thought into it’^42^ or that they were treated with respect, ‘I want them to treat me like a human’.^44^ Some CALD women felt they needed to educate HCP who were not providing culturally relevant information; ‘I am associated with the dietitian… but there is a slight gap here. So I try to convert everything from my side to present it to her’.^33^

### 3.6 Mental health challenges

#### 3.6.1 Concerns for baby

Women from both groups were concerned about the effect that GDM might have on their baby; ‘First of all I am very worried for the baby…that he will be affected by the diabetes’.^35^ Many women expressed anxiety about the discord between wanting to eat certain foods and not wanting to harm the baby; ‘I am hungry but I don’t want to eat now because I don’t know if I harm the baby’.^46^

#### 3.6.2 Anxiety, guilt and loss of control

Women from both groups expressed feelings of anxiety and guilt, ‘When I tested my blood sugar, if it was high, it made me very anxious’ and they felt loss of control over their pregnancy, ‘all my concentration is on …how much I eat, [and] walk… I don’t do anything else’.^27^ Women also expressed that they were ‘too scared to try anything else’^46^ at the risk of worsening their condition. Both groups rated the psychological impact of GDM as moderate with CALD women rating the impact with a score of 20.23/30 and non‐CALD women rating the impact with a score of 21.07/30 (Table 4).^18^

#### 3.6.3 Importance of well‐being

Women from both groups expressed that the experience of GDM made them realize the importance of being healthy, avoiding stress, and finding peace of mind. ‘Health means everything…I need to feel healthy… feel well… [to be] able to do everything’.^37^

### 3.7 Facilitators and barriers to support

#### 3.7.1 Emotional and family support

Women from both groups described their partners and mothers as valuable supporters, specifically regarding diet and exercise requirements, ‘He'll go and check online that what I'm having has the proper content of carbs in it, proper content of nutrition into it. So all these things that would have taken a lot of time for me, energy from me, and probably I would not have done.^33^ Women also described that seeing others with GDM made them feel relieved and less alone; When I see the other women with the same symptoms and problems, I felt relieved that there was somebody like me’.^32^

#### 3.7.2 Financial barriers

Culturally and linguistically diverse women did express that the GDM diet was more expensive than a normal diet. However, there was a unanimous experience that ‘Health is more important than money’.^35^

#### 3.7.3 Social and cultural challenges

Culturally and linguistically diverse women expressed that eating out and dining at friends' places was difficult due to diet restrictions; ‘it is difficult for me if my friends invite me they make a lot of food and it is difficult to weigh my bread and figure out how much (to eat) […] They don’t know about GDM, they just say just eat a little of this and a little of this’.^32^ Some CALD women expressed that they experienced stigma associated with GDM, finding that others thought it was ‘contagious’^44^ and that they needed to ‘make it secret’.^44^

## 4 DISCUSSION

This is the first systematic review to provide a thorough synthesis of the qualitative and quantitative literature that outlines the experiences of women with GDM from CALD and non‐CALD backgrounds. There were major similarities in experience of GDM between both groups of women. These included the burden of self‐management recommendations, their interactions with the healthcare system and mental health challenges. Such similarities highlight that GDM is universally a burdensome and stressful condition, irrespective of cultural background. However, the differences in the experience of women from CALD backgrounds were highlighted, in particular the cultural relevance of dietary recommendations being a unique burden experienced by CALD women. CALD women also found it more challenging to apply the blood glucose management advice, and they were more likely to express experiences with financial, social and cultural barriers The differences identified by this systematic review provide insights into the challenges associated with GDM diagnosis and management in CALD women.

Some experiences related to GDM were universal to both CALD and non‐CALD. Many women expressed that they experienced feelings of anxiety, guilt and a loss of control over their pregnancy. Specifically, women expressed concern about the long‐term health consequences that GDM might have for both themselves and their baby, and expressed that they did not want to try something new for fear of potential harm. Indeed, GDM is associated with higher rates of anxiety, antenatal and postnatal depression,^47^ with one study reporting a fourfold increase in risk of developing postpartum depression after having GDM.^48^ The findings in this review suggest that the negative psychological consequences of GDM are significant and ongoing for many women. Therefore, support for women with GDM should be directed towards validating the uncertainty and stressful nature of a new diagnosis in pregnancy. Further, HCPs should look towards providing tailored emotional and psychological resources for women with GDM.

Gestational diabetes mellitus education involves a huge increase in knowledge and a change in lifestyle for almost all women. Women find the self‐management requirements confusing and cumbersome, specifically blood glucose monitoring and carbohydrate counting. This resulted in women from both groups feeling overwhelmed. With respect to HCP interactions, women reported limited time for consultations, feeling threatened and being pressured into making decisions. Previous studies have reported on the negative impact that a paternalistic healthcare model can have on the patient‐HCP relationship.^49^ The lack of choice expressed by women in this systematic review highlights the importance of open dialogue and patient autonomy. The value of empowerment of women with GDM in self‐management has been previously reported; one study described increasing patient ‘self‐efficacy’ and their role in decision‐making as a method for improving patient autonomy.^50^ An increased focus on a shared decision‐making model for GDM management could assist HCPs to be more cognisant of the diverse needs of each woman. Furthermore, given the large number of women diagnosed with GDM each year it is paramount that HCPs have adequate time to address individual needs. The strategic use of group sessions, telehealth and pre‐recorded videos, specific to CALD and non‐CALD groups, could provide time‐efficient education, freeing up time for HCPs to spend more time addressing individual concerns. Another solution may be to promote GDM education for all pregnant women prior to diagnosis. Prior awareness may reduce the anxiety and shock experienced and improve the experience and interaction of women with the healthcare system.

The major difference in experience unique to CALD background women was the lack of culturally relevant dietary recommendations. Frustration at the standardized approach to food‐based recommendations was at the centre of CALD women's experience. A lack of specific and culturally relevant resources for CALD women with GDM has been identified as a long‐standing issue. Attempts to develop culturally sensitive dietary recommendations have been made; however, they have fallen short in addressing nuanced and specific requirements of each woman.^7^, ^45^ Specifically, the resources around dietary substitutions available to women with GDM by the Australian National Diabetes Services Schemes (NDSS) are broad and based heavily on a western diet, though pleasingly translated in a range of languages included Arabic, Bengali, Chinese (simplified and traditional), Hindi, Nepali, Punjabi, Turkish, Urdu and Vietnamese.^51^ Though effort has been made to include recommendations for a Chinese or Indian style cuisine, these recommendations remain generic and broad, with one woman from our review expressing that a dietary plan for Cantonese eating habits would not be relevant to patients from Northern China due to the heterogeneity across different regions.^45^ Increased education for HCPs and dietitians around culturally relevant dietary recommendations is required. Educational opportunities should be specific to the predominant cultural backgrounds in the relevant local health district. Additionally, a focus on community lead education, provided by CALD women, would complement the management of their heterogenous needs. A personalized, case‐by‐case approach to culturally sensitive dietary advice and culturally competent care is necessary if CALD women are expected to successfully implement dietary changes following GDM diagnosis.^46^

A strength of our study was that we were able to analyse the experiences of women with GDM originating from many different countries across the globe, including CALD women from East Asia (China/Korea/Japan), South East Asia (Filipino/Vietnamese), South Asia (Indian subcontinent), Central Asia (Middle East), Western Asia (Eastern European), Latin American, West Indian, African American, Hispanic, Appalachian, Pacific Islander, Moroccan and African, and non‐CALD women from Australia, Sweden, Canada, Denmark, Norway, the United States and the UK. The mixed methods approach provided a synthesis of the available evidence, not possible to the same degree in a single method's systematic review.^22^ Mixed method is particularly suited to describing the experiences of women with GDM from CALD backgrounds. Quantitative studies captured women's response to dietary recommendations, identifying that both groups required more knowledge of carbohydrate food substitutions in GDM.^19^ The qualitative studies highlighted the very different experience of women from CALD background compared to women from non‐CALD backgrounds in terms of frustration and relevance of food substitutions.^27^ Both findings are essential in improving direction of care, specifically for women from CALD backgrounds. Overall, the mixed‐methodology approach enabled qualitative and quantitative data to be included, therefore enabling a broad and deep perspective regarding the GDM experience.

The systematic review was limited by the quality of the studies included, and though all studies included data on CALD women's experience, not all studies included comparative data to a non‐CALD group. Therefore, the comparison of CALD and non‐CALD experiences was assessed across different studies. Further, due to the variability in the outcome measures used in the four quantitative papers obtained, meta‐analysis was not possible. Additionally, the experiences of a specific subset of women, such as Caucasian women living in Asia, has been poorly explored in the literature. Therefore, findings may not be generalisable to all women with GDM as many CALD and non‐CALD ethnicities were not represented in the existing studies. An additional limitation of our study was that publications were restricted to English language. CALD can be locally defined and different from setting to setting, such that there was the potential to have missed original studies in languages other than English. Furthermore, quality assessment was only completed by 1 reviewer; therefore, there is a potential risk of error.

The contribution of this review to the literature highlights the need for a personalized approach to GDM management, as there is no appropriate ‘one size fits all’ model. Future studies should include interventional strategies to identify the benefits of early, targeted GDM education for pregnant women prior to diagnosis. GDM education should be appropriate for all levels of health literacy and languages. Additionally, HCPs should be required to ensure acknowledgement of the sensitivities around GDM diagnosis and management, and ensure that culturally relevant dietary information is provided, particularly to women from CALD backgrounds. Models of care for GDM should focus on a personalized approach and shared decision‐making. A call for further research and continued optimisation in this area is necessary to improve the experiences of all women with GDM.

## 5 CONCLUSION

Overall, the findings from this systematic review highlight multiple similarities and some key differences in the experiences of GDM in women from CALD versus non‐CALD backgrounds. The review provides valuable insights into how HCPs and policy makers could implement change. Implementation of the findings from this review will enhance the patient experience of GDM for all women. Nonetheless, our study calls for regular audit to be conducted at a local health district level to better understand the unique experiences of women with GDM in communities that differ in ethnicity, socioeconomic background and educational level, such that change enacted can be specific, tailored and successful.

## AUTHOR CONTRIBUTIONS

SG conceived the study. CH carried out the literature search, assessed the risk of bias, completed the data synthesis with support from RL, CE, and SG and wrote the manuscript with support from SG. CH and RL independently screened the titles, abstracts, and full texts and extracted the data with support from TW and SG. All authors revised the manuscript.

## CONFLICT OF INTEREST STATEMENT

The authors have no actual or potential conflicts of interest.

## ETHICS STATEMENT

No ethics approval was obtained as no original human data were included in this manuscript.

## Supporting information


Appendix S1:

## Data Availability

All data extracted and analysed are included within the manuscript.
