# Supplementary material for: The experiences of women from culturally and linguistically diverse backgrounds with gestational diabetes mellitus: A mixed methods systematic review
Source: Endocrinol Diabetes Metab. 2023 May 4;6(4):e421. doi: 10.1002/edm2.421 (PMC10335617; doi:10.1002/edm2.421)
Supplement: Supplementary file 1 — Appendix S1: [file EDM2-6-e421-s001.pdf]

Supplementary Material

Supplementary Material 1. Prospero

https://www.crd.york.ac.uk/prospero/display_record.php?RecordID=148779&VersionID=1296444

Supplementary Material 2. Prisma

PRISMA-P (Preferred Reporting Items for Systematic review and Meta-Analysis Protocols) 2015 checklist: recommended items to address in a systematic review protocol*

Section and topic	Item No	Checklist item
**ADMINISTRATIVE INFORMATION**		
Title:		The experiences of culturally and linguistically diverse background women with gestational diabetes mellitus: A mixed methods systematic review
Identification	1a	This is a protocol of a systematic review
Update	1b	This is a new Systematic Review
Registration	2	Registered via PROSPERO 2020 registration number: CRD42020148779<br>Available from: https://www.crd.york.ac.uk/prospero/display_record.php?ID=CRD42020148779
Authors:		
Contact	3a	Claire E. Haigh Medical Student, Royal North Shore Hospital, Northern Clinical School, University of Sydney. St Leonards NSW 2065. Chai3241@uni.sydney.edu.au<br>Sarah Glastras, Staff Specialist in Endocrinology, Royal North Shore Hospital. St. Leonards NSW 2065, Sarah.glastras@sydney.edu.au
Contributions	3b	Claire E. Haigh will write the protocol as the primary reviewer. Rachel Lau will be the second reviewer.
Amendments	4	No previous amendments
Support:		
Sources	5a	Indicate sources of financial or other support for the review
Sponsor	5b	Provide name for the review funder and/or sponsor
Role of sponsor or funder	5c	Describe roles of funder(s), sponsor(s), and/or institution(s), if any, in developing the protocol
**INTRODUCTION**		
Rationale	6	Gestational Diabetes Mellitus (GDM) is the diagnosis of new onset glucose intolerance during the second or third trimester of pregnancy (1). Poorly managed GDM can lead to adverse maternal and foetal outcomes, such as large birth weight, delivery complications and increased risk of maternal type 2 diabetes mellitus (2- 4). In 2016-17, the incidence of GDM in Australia was 15%, with a predominance towards women from culturally and linguistically diverse (CALD) backgrounds, such as Asia, India, and the Middle East (5, 6). The literature has shown that high levels of stress, anxiety and confusion are experienced by women with GDM (7-9). Poor comprehension of diagnosis and management of GDM has been identified as a key factor for increased stress (10, 11). Additionally, time constraints, lower educational standards and varying cultural beliefs surrounding food traditions have been identified as confounders for differences in experience (10, 12). Therefore, the aim of this literature review is to

		summarise what is known about the experiences of CALD background women with GDM, to highlight areas where synthesis of their experience is needed and to suggest what future investigation is needed.<br><br>1. American Diabetes A. 2. Classification and Diagnosis of Diabetes: Standards of Medical Care in Diabetes—2021. Diabetes Care. 2020;44(Supplement_1):S15-S33.<br>2. HAPO. Hyperglycemia and Adverse Pregnancy Outcomes. New England Journal of Medicine. 2008;358(19):1991-2002.<br>3. Nankervis A, Conn J. Gestational diabetes mellitus. Australian Journal for General Practitioners. 2013;42:528-31.<br>4. Vounzoulaki E, Khunti K, Abner SC, Tan BK, Davies MJ, Gillies CL. Progression to type 2 diabetes in women with a known history of gestational diabetes: Systematic review and meta-analysis. BMJ (Online). 2020;369:m1361-m.<br>5. Health AIo, Welfare. Incidence of gestational diabetes in Australia. Canberra: AIHW; 2019.<br>6. Yuen L, Wong VW. Gestational diabetes mellitus: Challenges for different ethnic groups. World Journal of Diabetes. 2015;6(8):1024-32.<br>7. Draffin CR, Alderdice FA, McCance DR, Maresh M, Harper MR, McSorley O, et al. Exploring the needs, concerns and knowledge of women diagnosed with gestational diabetes: A qualitative study. Midwifery. 2016;40:141-7.<br>8. Persson M, Winkvist A, Mogren I. 'From stun to gradual balance'- women's experiences of living with gestational diabetes mellitus. Scandinavian journal of caring sciences. 2010;24(3):454-62.<br>9. Carolan-Olah M, Duarte-Gardea M, Lechuga J, Salinas-Lopez S. The experience of gestational diabetes mellitus (GDM) among Hispanic women in a U.S. border region. Sexual & Reproductive Healthcare. 2017;12:16-23.<br>10. Carolan M, Steele C, Margetts H. Knowledge of gestational diabetes among a multi-ethnic cohort in Australia. Midwifery. 2010;26(6):579-88.<br>11. Devsam BU, Bogossian FE, Peacock AS. An interpretive review of women's experiences of gestational diabetes mellitus: Proposing a framework to enhance midwifery assessment. Women and Birth. 2013;26(2):e69-e76.<br>12. Carolan M, Steele C, Margetts H. Attitudes towards gestational diabetes among a multiethnic cohort in Australia. J Clin Nurs. 2010;19(17-18):2446-53.
Objectives	7	To summarise the experiences of CALD background women with GDM and how their experiences are similar and different to that of non-CALD women.<br>To suggest future investigations and strategies that could be implemented to optimise the GDM experience for CALD women.
**METHODS**		
Eligibility criteria	8	Specify the study characteristics (such as PICO, study design, setting, time frame) and report characteristics (such as years considered, language, publication status) to be used as criteria for eligibility for the review<br>The aim was to answer our specific PICO question:

		P – Culturally and Linguistically Diverse (born in a different country) background women with Gestational Diabetes Mellitus<br>I – The health care system, hospitals<br>C - English speaking/ Caucasian/native born women with gestational diabetes<br>O- Perspective and Experience<br>o Diagnosis, interaction with health care system, GDM: education, dietary, exercise, and blood glucose control, mental health; anxiety and stress, support; emotional or family.<br><br>Study types may include:<br>• Randomised control trials<br>• Clinical trials<br>• Cohort studies<br>• Cross-sectional studies<br>• Unselected case series<br><br>The specific inclusion criteria include a clear GDM definition, women with a new diagnosis of GDM or history of GDM, women from CALD background, and qualitative or quantitative data on the woman's perspective or experience of GDM during any stage pregnancy<br><br>The specific exclusion criteria included studies that had women with a previous diagnosis of diabetes mellitus (T1DM or T2DM) prior to pregnancy, did not differentiate between GDM and T2DM, did not separate CALD from non-CALD data, focused on outcome, incidence/prevalence, or risk factors for GDM or post-partum experience, or was not a primary study
Information sources	9	Describe all intended information sources (such as electronic databases, contact with study authors, trial registers or other grey literature sources) with planned dates of coverage<br><br>The initial search strategy will be performed in MEDLINE and then adapted for each of the different databases. The databases search includes MEDLINE, EMBASE, PsycINFO, Scopus, WOS and CINAHL, searched from inception to February 2022. The key terms developed are around the two areas of: i) gestational diabetes mellitus and ii) culturally and linguistically diverse background women.
Search strategy	10	Present draft of search strategy to be used for at least one electronic database, including planned limits, such that it could be repeated<br>MEDLINE, EMBASE, PsycINFO, Scopus, WOS and CINAHL,<br>Database: Ovid MEDLINE(R) ALL <1946 to February 02, 2022><br>Search Strategy:

----

1 Diabetes, Gestational/ (13068)
2 Gestation* Diabet*.mp. (18296)
3 cultural diversity/ (12424)
4 CALD.mp. (447)
5 ((cultural* or linguistic* or language*) adj3 divers*).mp. (17742)
6 nesb.mp. (83)
7 "emigrants and immigrants"/ or undocumented immigrants/ (14427)
8 (migrant* or immigrant*).mp. (55115)
9 Minority Groups/ (16021)
10 exp Ethnic Groups/ (167208)
11 ((minority or ethnic or divers* or non-english) adj2 (culture* or group* or minorit* or speak*)).mp. (182556)
12 ((first or native or mother or foreign) and (language* or tongue*)).mp. (45422)
13 ((foreign or overseas) adj3 born).mp. (4258)
14 (first adj3 generation*).mp. (20641)
15 Refugees/ (11854)
16 refugee*.mp. (16404)
17 1 or 2 (20292)
18 3 or 4 or 5 or 6 or 7 or 8 or 9 or 10 or 11 or 12 or 13 or 14 or 15 or 16 (397737)
19 17 and 18 (847)
20 limit 19 to humans (773)
21 limit 19 to (english language and humans) (760)
22 limit 19 to (english language and humans and yr="2000 -Current") (686)
23 limit 19 to (humans and yr="2000 -Current") (698)

****

Study records:		
Data management	11a	Describe the mechanism(s) that will be used to manage records and data throughout the review<br>Endnote and Covidence will be used to remove duplicated articles. Two reviewers will use Covidence to title, abstract and full text screen the obtained studies. JBI critical appraisal checklists for analytical cross-sectional studies and qualitative research will be used to assess the risk of bias and methodological quality of the studies in this review. Data analysis: for qualitative data analysis the software, nVivo, will be used to draw out themes. While quantitative data will be qualitized into a mutually compatible qualitative format via narrative description.

Selection process	11b	State the process that will be used for selecting studies (such as two independent reviewers) through each phase of the review (that is, screening, eligibility, and inclusion in meta-analysis)<br>Two independent reviewers, CH and RL will independently screen all records according to title, abstract and full text papers against the eligibility criteria. Any discrepancies will be discussed by CH and RL, and if consensus cannot be reach, taken to the third reviewer SG.
Data collection process	11c	Describe planned method of extracting data from reports (such as piloting forms, done independently, in duplicate), any processes for obtaining and confirming data from investigators<br>De-duplication was initially conducted in Endnote. Further de-duplication will occur in Covidence. The two primary reviewers (CH and RL) will independently screen the titles, abstracts and full texts using Covidence. Studies will be excluded if they do not meet the eligibility criteria.
Data items	12	List and define all variables for which data will be sought (such as PICO items, funding sources), any pre-planned data assumptions and simplifications<br><br>1. Authors details and country or origin<br><br>2. Title of article<br><br>3. Types of participants:<br>A) CALD women with reported experiences for GDM management (must have been diagnosed with GDM).<br>B) Native born women with reported experiences for GDM management (must have been diagnosed with GDM).<br><br>4. Number of participants in each category<br>A) N/A<br>B) N/A<br><br>6. Funding sources<br><br>7. Characteristics of participants<br>A) Current or Previous GDM<br>B) CALD background: East Asia (China/Korea/Japan), South East Asia (Filipino/Vietnamese), South Asia (Indian subcontinent), Central Asia (Middle East), Western Asia (Eastern European), Latin American, West Indian, African American, Hispanic, Appalachian, First Nations/Aboriginal, Caucasian, Black, Pacific Islander or Other<br>C) GDM method of diagnosis: 75g OGTT at 24-28 weeks, Clinical/clinician decision, Blood test (other than OGTT), Unspecific or Other

D) Socioeconomic background considered: yes/no, and level of education (Low = high school, medium = university/College, high = post grad/masters)
E) Inclusion criteria
F) Exclusion criteria

9. Data collection and measurement tools-
A) Aim of study
B) Study design: randomised controlled trial, non-randomised experimental study, Cohort study, Cross sectional study, case control study, qualitative research, case series, case report, text and opinion, Other
C) Sampling context: questionnaire (yes/no), questionnaire (pictorial), questionnaire (lichet scale), semi-structured Interview, unstructured Interview and unclear/unspecified
D) Data collection tool used: Thematic analysis tool (nVivo etc) or No thematic analysis
E) **Method of recruitment of participant**

1. Clinic patients
2. Phone
3. Mail
4. Voluntary
5. Clinical Trial
6. Other

10. Author definitions –
A) Gestational Diabetes Mellitus
B) Cultural and linguistically diverse
C) Experience

11. Details of Interventions (e.g. management of GDM, health care system and comparators).

12. Outcomes/results (specify each outcome)
A) Diagnosis: response and understanding
B) Access to care and interaction with HCP
C) Self-management: education, dietary, exercise, and blood glucose control
D) Mental health: anxiety, stress, or burden
E) Support: emotional or otherwise

Outcomes and prioritization	13	List and define all outcomes for which data will be sought, including prioritization of main and additional outcomes, with rationale<br><br>The primary outcome of this systematic review is to summarise the experiences of CALD background women with GDM and how their experiences are similar and different to that of non-CALD women.<br><br>The secondary outcome of this review is to suggest future investigations and strategies that could be implemented to optimise the GDM experience for CALD women.
Risk of bias in individual studies	14	Describe anticipated methods for assessing risk of bias of individual studies, including whether this will be done at the outcome or study level, or both; state how this information will be used in data synthesis.<br><br>For every included study CH will independently do a quality appraisal using The JBI critical appraisal checklists for analytical cross-sectional studies and qualitative research were used to assess the risk of bias and methodological quality of the studies in this review. The scoring will be determined as: yes, no, unclear, or not applicable.
Data synthesis	15a	Describe criteria under which study data will be quantitatively synthesised<br><br>The authors will look for quantitative and qualitative assessments of “experiences” in CALD women in the studies, and synthesise similar measured outcomes together. However, it should be noted that it will be difficult to find many studies that measure the same outcomes in the inclusion criteria, such the quantitative data will be qualitized into a mutually compatible qualitative format of narrative description.
	15b	If data are appropriate for quantitative synthesis, describe planned summary measures, methods of handling data and methods of combining data from studies, including any planned exploration of consistency (such as I^2, Kendall’s τ)<br><br>When multiple studies are reporting the same outcomes, a meta-analysis will be performed in R. Where applicable, heterogeneity of effect for studies within a meta-analysis will be assessed with the I2 statistic.
	15c	Describe any proposed additional analyses (such as sensitivity or subgroup analyses, meta-regression)<br><br>A subgroup analysis is not proposed<br><br>If quantitative synthesis is not appropriate, describe the type of summary planned

		If the studies do not report the same outcomes, the results of each study will be interpreted qualitatively. Transformation of the quantitative data into a mutually compatible qualitative format will be done to compare results and produce more complex analysis.
Meta-bias(es)	16	Specify any planned assessment of meta-bias(es) (such as publication bias across studies, selective reporting within studies)<br><br>N/A
Confidence in cumulative evidence	17	Describe how the strength of the body of evidence will be assessed (such as GRADE)<br><br>The quality of evidence applied to the outcomes from this study can be assessed using the GRADE (Grading of Recommendations, Assessment, Development and Evaluations) quality rating.

*** It is strongly recommended that this checklist be read in conjunction with the PRISMA-P Explanation and Elaboration (cite when available) for important clarification on the items. Amendments to a review protocol should be tracked and dated.** **The copyright for PRISMA-P (including checklist) is held by the PRISMA-P Group and is distributed under a Creative Commons Attribution Licence 4.0.**

From: Shamseer L, Moher D, Clarke M, Ghersi D, Liberati A, Petticrew M, Shekelle P, Stewart L, PRISMA-P Group. Preferred reporting items for systematic review and meta-analysis protocols (PRISMA-P) 2015: elaboration and explanation. BMJ. 2015 Jan 2;349(jan02 1):g7647.

Supplementary Material 3. Search strategy

Database: Ovid MEDLINE(R) ALL <1946 to February 02, 2022>

Search Strategy:

----

1 Diabetes, Gestational/ (13068)

2 Gestation* Diabet*.mp. (18296)

3 cultural diversity/ (12424)

4 CALD.mp. (447)

5 ((cultural* or linguistic* or language*) adj3 divers*).mp. (17742)

6 nesb.mp. (83)

7 "emigrants and immigrants"/ or undocumented immigrants/ (14427)

8 (migrant* or immigrant*).mp. (55115)

9 Minority Groups/ (16021)

10 exp Ethnic Groups/ (167208)

11 ((minority or ethnic or divers* or non-english) adj2 (culture* or group* or minorit* or speak*)).mp. (182556)

12 ((first or native or mother or foreign) and (language* or tongue*)).mp. (45422)

13 ((foreign or overseas) adj3 born).mp. (4258)

14 (first adj3 generation*).mp. (20641)

15 Refugees/ (11854)

16 refugee*.mp. (16404)

17 1 or 2 (20292)

18 3 or 4 or 5 or 6 or 7 or 8 or 9 or 10 or 11 or 12 or 13 or 14 or 15 or 16 (397737)

19 17 and 18 (847)

20 limit 19 to humans (773)

21 limit 19 to (english language and humans) (760)

22 limit 19 to (english language and humans and yr="2000 -Current") (686)

23 limit 19 to (humans and yr="2000 -Current") (698)

****

Database: Embase Classic+Embase <1947 to 2022 February 02>

Search Strategy:

----

1 pregnancy diabetes mellitus/ or maternal diabetes mellitus/ (43391)

2 Gestation* Diabet*.mp. (29055)

3 cultural diversity/ (2572)

4 CALD.mp. (719)

5 ((cultural* or linguistic* or language*) adj3 divers*).mp. (10159)

6 nesb.mp. (113)

7 migrant/ or emigrant/ or forced migrant/ or immigrant/ or migrant worker/ (30591)

8 (migrant* or immigrant*).mp. (61017)

9 minority group/ or integration/ (23472)

10 ethnic group/ (78565)

11 ((minority or ethnic or divers* or non-english) adj2 (culture* or group* or minorit* or speak*)).mp. (222301)

12 ((first or native or mother or foreign) and (language* or tongue*)).mp. (63724)

13 ((foreign or overseas) adj3 born).mp. (5318)

14 (first adj3 generation*).mp. (28262)

15 exp refugee/ (15365)

16 refugee*.mp. (18191)

17 1 or 2 (46433)

18 3 or 4 or 5 or 6 or 7 or 8 or 9 or 10 or 11 or 12 or 13 or 14 or 15 or 16 (391001)

19 17 and 18 (1218)

20 limit 19 to human (1167)

21 limit 19 to (human and english language) (1134)

22 limit 19 to (human and english language and yr="2000 -Current") (1059)

23 limit 19 to (human and yr="2000 -Current") (1090)

****

Database: APA PsycInfo <1806 to January Week 5 2022>

Search Strategy:

----

1 gestational diabetes/ (247)

2 Gestation* Diabet*.mp. (681)

3 cultural diversity/ (2222)

4 CALD.mp. (186)

5 ((cultural* or linguistic* or language*) adj3 divers*).mp. (16536)

6 English as Second Language/ (8051)

7 nesb.mp. (40)

8 exp inclusion/ (9136)

9 (migrant* or immigrant*).mp. (40256)

10 minority groups/ or exp "race and ethnic discrimination"/ or minority stress/ (23355)

11 "racial and ethnic groups"/ or cross cultural communication/ or cross cultural differences/ or cross cultural psychology/ or exp cross cultural treatment/ or cultural sensitivity/ or ethnic diversity/ or ethnic values/ or interracial interactions/ or majority groups/ or multiculturalism/ or exp "racial and ethnic attitudes"/ or exp "racial and ethnic differences"/ or exp racial bias/ or racial disparities/ or racial identity/ or exp sociocultural factors/ (195441)

12 ((minority or ethnic or divers* or non-english) adj2 (culture* or group* or minorit* or speak*)).mp. (95996)

13 ((first or native or mother or foreign) and (language* or tongue*)).mp. (80720)

14 ((foreign or overseas) adj3 born).mp. (2386)

15 (first adj3 generation*).mp. (6193)

16 refugees/ or immigration/ (30534)

17 refugee*.mp. (10987)

18 1 or 2 (681)

19 3 or 4 or 5 or 6 or 7 or 8 or 9 or 10 or 11 or 12 or 13 or 14 or 15 or 16 or 17 (378307)

20 18 and 19 (90)

21 limit 20 to human (88)

22 limit 20 to (human and english language) (87)

23 limit 20 to (human and english language and yr="2000 -Current") (86)

24 limit 20 to (human and yr="2000 -Current") (87)

****

Scopus 618

“Gestation* Diabet*”

AND

(((cultural* or linguistic* or language*) W/3 divers*) OR CALD OR nesb OR ((minority or ethnic or divers* or non-english) W/2 (culture* or group* or minorit* or speak*)) OR ((first or native or mother or foreign) and (language* or tongue*)) OR ((foreign or overseas) W/3 born) OR (first W/3 generation*) or refugee* OR migrant* or immigrant*)

(TITLE-ABS-KEY ("Gestation* Diabet*") AND TITLE-ABS-KEY ((((cultural* OR linguistic* OR language*) W/3 divers*) OR cald OR nesb OR ((minority OR ethnic OR divers* OR non-english) W/2 (culture* OR group* OR minorit* OR speak*)) OR ((first OR native OR mother OR foreign) AND (language* OR tongue*)) OR ((foreign OR overseas) W/3 born) OR (first W/3 generation*) OR refugee* OR migrant* OR immigrant*))) AND PUBYEAR > 1999 AND (LIMIT-TO (LANGUAGE , "English")) AND (LIMIT-TO (EXACTKEYWORD , "Human"))

WOS core collection 228

“Gestation* Diabet*” (All Fields)

AND

(((cultural* or linguistic* or language*) N3 divers*) OR CALD OR nesb OR ((minority or ethnic or divers* or non-english) N2 (culture* or group* or minorit* or speak*)) OR ((first or native or mother or foreign) and (language* or tongue*)) OR ((foreign or overseas) N3 born) OR (first N3 generation*) or refugee* OR migrant* or immigrant*) (All Fields)

CINAHL 226

"AB “Gestation* Diabet*”

AND

AB ((((cultural* or linguistic* or language*) W3 divers*) OR CALD OR nesb OR ((minority or ethnic or divers* or non-english) W2 (culture* or group* or minorit* or speak*)) OR ((first or native or mother or foreign) and (language* or tongue*)) OR ((foreign or overseas) W3 born) OR (first W3 generation*) or refugee* OR migrant* or immigrant*))

Supplementary Material 4. JBI critical appraisal checklists for analytical cross-sectional studies

Below
